# The management of patients with predominant negative symptoms in Slovakia: A 1-year longitudinal, prospective, multicentric cohort study

**DOI:** 10.1192/j.eurpsy.2024.1757

**Published:** 2024-05-23

**Authors:** Jozef Dragasek, Zsofia Borbala Dombi, Károly Acsai, Viktor Dzurilla, Ágota Barabássy

**Affiliations:** 1 1st Department of Psychiatry, Pavol Jozef Safarik University, Faculty of Medicine and University Hospital of Louis Pasteur, Košice, Slovakia; 2 Global Medical Division, Gedeon Richter Plc., Budapest, Hungary; 3 Ceva Animal Health, Ceva-Phylaxia, Budapest, Hungary; 4 Gedeon Richter Slovakia, Bratislava, Slovakia

**Keywords:** antipsychotic medication, negative symptoms, observational study, schizophrenia

## Abstract

**Background:**

Predominant negative symptoms (PNSs) in schizophrenia can affect the patients’ psychosocial functioning immensely and are less responsive to treatment than positive symptoms.

**Aims:**

The aim of the study was to observe negative symptoms and psychosocial functioning in PNS schizophrenia patients and to understand whether PNS can be improved and with what treatment strategies.

**Methods:**

This was a 1-year, prospective, multicentric cohort study conducted in Slovakia. Adult outpatients with diagnosis of schizophrenia according to ICD-10 and PNS evaluated using the criteria by the European Psychiatric Association’s (EPA) guidance were included. Change in negative symptoms, functionality, and treatment patterns were observed. Treatment effectiveness was evaluated using the modified Short Assessment of Negative Domain (m-SAND), the Self-evaluation of Negative Symptoms (SNS) scale, the Personal and Social Performance (PSP) scale, and the Clinical Global Impression – Severity (CGI-S) and the Clinical Global Impression – Improvement (CGI-I) scales. Least-squares (LS) means were calculated for the change from baseline to final visit for the outcomes.

**Results:**

The study included 188 patients. Functionality improved as, by the end of the study, fewer patients were unemployed (53%) and more worked occasionally (21%). PNS improved significantly according to both physicians and patients (LS mean change from baseline in m-SAND total score: -10.0 (p-value <0.0001)). Most patients received polytherapy throughout the study. Cariprazine was utilized most (20% monotherapy and 76% polytherapy). Only a few patients discontinued treatment due to adverse drug reactions.

**Conclusions:**

With the right treatment strategy, it is possible to achieve improvement in PNS and everyday functioning in schizophrenia outpatients.

## Introduction

Schizophrenia is a chronic psychiatric disorder affecting approximately 1% of the general population [1] and is one of the most disabling health conditions in the world [2]. It is also associated with significant financial and health burdens; patients with schizophrenia have increased risk of non-communicable diseases as well as higher mortality rates [3, 4]. In addition, due to functional impairment and the costs of treatment and care, there is a major loss of productivity, affecting not only the patients themselves, but their caregivers too [5]. A recent epidemiological study examining the burden of schizophrenia in Central and Eastern Europe (CEE) found 14% of Slovakian schizophrenia patients to be unemployed and 63% to live on a disability pension [5]. In addition, on average, 4% of caregivers had to stop working to take care of their relatives [5].

Characterized by a wide range of symptoms, schizophrenia is a multidimensional disorder [6]. According to recent conceptualizations, negative symptoms are comprised of five constructs, the so-called 5As: anhedonia, alogia, avolition, asociality, and affective flattening [7–9]. If the severity of negative symptoms exceeds that of the positive symptoms, the patient is called a predominant negative symptom (PNS) schizophrenia patient [7]. Negative symptoms can be primary or secondary depending on their root cause: while primary negative symptoms are intrinsic to the disorder, secondary negative symptoms are triggered by other factors such as adverse effects of treatment or other symptom domains [7].

Negative symptoms are well-known to affect daily functioning and quality of life (QoL) immensely [7, 10–12]. For instance, in a 3-year study with 17,384 outpatients from 37 countries, QoL was found to correlate with negative symptoms more than with positive symptoms [12]. Furthermore, a recent study by D’Anna et al. evaluating the relationship between negative symptoms and daily time use found that patients with more negative symptomatology spent more time with non-productive activities compared to patients with milder symptoms [11].

Schizophrenia is primarily treated with antipsychotic medications [13]. According to a recent study in Slovakia, the first-line treatment of schizophrenia based on expert opinion is risperidone (36%), olanzapine (28%), and quetiapine (13%) [13]. Having a more balanced safety profile, second-generation antipsychotics are preferred over first-generation ones (~70% vs 30%) in Slovakia in general [13]. In terms of negative symptoms, a recent proposal by Cerveri et al. recommends cariprazine as a first-line medication due to its partial agonist effect on the dopamine D3-D2 receptors [14, 15]. Indeed, according to a review involving 17 experts from the CEE region, the Cerveri treatment algorithm has been adapted in Slovakia as well [16].

The aim of the present cohort study was twofold: first, to observe the negative symptom domain and its association with psychosocial functioning in patients with PNS and the typical treatment patterns in Slovakia, and second, to observe whether PNS can improve in an outpatient setting throughout a 1-year treatment period and with what pharmacological and non-pharmacological treatment strategies.

## Methods

### Study design

This was a longitudinal, prospective, multicentric cohort study conducted in 20 sites in Slovakia. The study duration was 1 year, with three visits after baseline at 3, 6, and 12 months.

### Patient characteristics

The inclusion criteria were the following: adult outpatients (between ages 18 and 65) with a schizophrenia diagnosis according to the International Classification of Diseases 10^th^ edition (ICD-10) who exhibited predominant negative symptoms according to the European Psychiatric Association’s (EPA) guidance were included in the study [17]. The EPA guidance suggests the presence of at least moderate severity of at least two symptoms, which was evaluated and decided by the doctors based on the patient’s anamnesis [17]. Patients with comorbid neurological disorders were excluded. The cohort study received approval by the Ethics Committee of the Košice Self-Governing Region (3618/2020/ODDZ-07169), and informed written consent was obtained from all participants. The study complies with the Declaration of Helsinki.

### Measures

Epidemiological measures were general patient characteristics (sex, age, duration of illness, comorbidities), changes in the frequency of functionality outcomes (employment status, disability status, and disorder insight), changes in the frequency of primary and secondary negative symptoms, and changes in the frequency of treatment patterns (frequency of monotherapy, polytherapy and non-pharmacotherapy) throughout the 1-year observational period. Primary and secondary negative symptoms were differentiated using a structured interview based on the guidance provided by the EPA [17]. Insight was defined as *“a person’s capacity to understand the nature, significance, and severity of his or her own illness”* [18], and whether a patient had full, partial, or no insight was determined by the physician based on the clinical interview.

The effectiveness of the different treatment strategies was assessed via the modified Short Assessment of Negative Domain (m-SAND) scale, the Self-evaluation of Negative Symptoms (SNS) scale [19], the Personal and Social Performance (PSP) scale [20], and the Clinical Global Impression – Severity (CGI-S) and the Clinical Global Impression – Improvement (CGI-I) scales [21]. Given the nature of the study, safety parameters and adverse events were monitored and addressed as in a routine clinical setting.

#### m-SAND scale

The original SAND was utilized in a Latvian observational study evaluating the effectiveness of cariprazine in predominant negative symptom patients [22]. The SAND is an anamnesis-based scale that is composed of seven items: two positive items (delusions and hallucinations), which make the SAND positive subscale (SAND-P), and five negative items (anhedonia, alogia, avolition, asociality, and affective flattening), which make the SAND negative subscale (SAND-N) [22]. Each item is rated from 0 to 6 (not observed, minimal, mild, moderate, moderately severe, severe, and extreme). The SAND was chosen due to its simplicity and ability to capture all constructs of the negative symptom domain; however, the rating was modified since it is highly difficult to differentiate between “minimal” and “mild” severities. Therefore, the m-SAND includes the same items, but it is rated from 0 to 5 (not observed, mild, moderate, moderately severe, severe, and extreme). The m-SAND scale can be found in the Supplementary material.

#### SNS scale

The SNS scale is a self-administered questionnaire that measures the five subdomains of negative symptoms (the 5As) in schizophrenia and schizoaffective disorder [19]. Being a self-administered questionnaire, SNS is an easily understandable instrument for patients with schizophrenia that provides meaningful information for clinicians regarding the patients’ own perception of their negative symptoms [19]. Thus, the SNS can complement observer ratings of negative symptoms as well as increase patient engagement.

#### PSP scale

The PSP scale is a clinical tool used to measure the routine social functioning of patients with psychiatric disorders [20]. It measures four areas of social and individual performance independently of symptomatology: socially useful activities, personal and social relationships, self-care, and disturbing and aggressive behaviours [20]. The PSP is a useful tool for providing additional valuable information when evaluating social functioning related to schizophrenia and the effectiveness of the treatment [23].

#### CGI-S and CGI-I scales

The CGI scale provides an overall clinician-determined summary measure regarding the severity of illness (CGI-S) and improvement (CGI-I) in patients with psychiatric disorders [21]. The CGI is rated on a 7-point scale [21]. It is considered to be a widely accepted tool that synthesizes the clinician’s impression of the global illness state of the patient [21].

##### Statistical analyses

Epidemiologic measures were summarized using descriptive statistics in percentages, means, and standard deviations. Least-squares (LS) means were calculated for the change from baseline to final visit for the effectiveness measures (m-SAND, SNS, PSP, and CGI-S) using a mixed model for repeated measures (MMRM). Bland–Altman agreement plots were created to compare how clinicians (m-SAND-N) versus how patients (SNS) rated negative symptoms. All analyses were conducted using Statistical Analysis Software (SAS).

## Results

### Epidemiologic measures

#### Patient characteristics

Baseline patient characteristics are summarized in Table 1. The mean age of the 188 patients who were included in the cohort study was 39.8, and 64.9% of them was men. The mean duration of illness was 12 years, and most of the cohort was diagnosed with paranoid schizophrenia (51.6%). Patients exhibited both psychiatric and somatic comorbidities such as depression (13.3%), substance abuse disorder (11.7%), and personality disorder (8.0%), as well as hypertension (10.6%), obesity (10.1%), and hyperlipidaemia (5.3%). During the 12-month observational period, 148 patients stayed in the cohort study.

**Table 1.** Patient characteristics

Population	
Safety population, *n* (%)	188 (100)
Demographics	
Age, mean (SD), y	39.8 (10.8)
Males, *n* (%)	122 (64.9)
Schizophrenia characteristics	
Duration of illness, mean (SD), y	12.0 (9.0)
Schizophrenia diagnosis, *n* (%)	
Paranoid schizophrenia	97 (51.6)
Residual schizophrenia	36 (19.1)
Undifferentiated schizophrenia	24 (12.7)
Simple schizophrenia	17 (9.0)
Other type of schizophrenia	14 (7.4)
Comorbidities	
Psychiatric comorbidity, *n* (%)	
Depression	25 (13.3)
Substance abuse	22 (11.7)
Personality disorder	15 (8.0)
Somatic comorbidity, *n* (%)	
Hypertension	20 (10.6)
Obesity	19 (10.1)
Hyperlipidaemia	10 (5.3)

#### Functionality and insight

At baseline, most patients were unemployed (63.8%), worked occasionally (10.6%), or part-time (11.2%) as displayed in Table 2. At the end of the 12-month observation, only 53.4% were unemployed and more patients worked occasionally (20.9) or part-time (12.8%). The disability status, on the other hand, increased from 76.1% to 83.3%. In terms of disorder insight, at baseline, most patients had partial (70.2%) or full (20.2%) insight, while around 10% of patients had no insight at all. By the end of the observational period, 53.4% had partial, 44.6% full, and 0.2% no insight.

**Table 2.** Functionality and insight

	Baseline (*n* = 188)	Final visit^a^ (*n* = 148)
Employment status, *n* (%)		
Full–time job	17 (9.0)	10 (6.8)
Part–time job	21 (11.2)	19 (12.8)
Occasionally working	20 (10.6)	31 (20.9)
Unemployed	120 (63.8)	79 (53.4)
Student	5 (2.7)	4 (2.7)
Prisoner	5 (2.7)	5 (3.4)
Disability, *n* (%)		
No disability	44 (23.4)	23 (15.5)
Disability due to psychiatric illness	143 (76.1)	124 (83.8)
Disability due to non–psychiatric illness	1 (0.05)	1 (0.07)
Insight, *n* (%)		
Full insight	38 (20.2)	66 (44.6)
Partial insight	132 (70.2)	79 (53.4)
No insight	18 (9.6)	3 (0.2)

a 12 months from baseline.

#### Primary and secondary negative symptoms

All patients had primary negative symptoms, both at baseline and at the end of the study Table 3. At baseline, 93% of patients had blunted affect, 87% apathy, 82% anhedonia, 76% asociality, and 53% alogia. After one year, most patients still experienced affective blunting (93%); nonetheless, the other aspects of negative symptomatology improved: only 62% of the patients had apathy, 56% anhedonia, 50% asociality, and 38% alogia. In addition to primary negative symptoms, a significant proportion of patients also had secondary negative symptoms (56%) due to affective symptoms (37%), positive symptoms (26%), and adverse drug reactions (21%) at baseline. Similar to primary negative symptoms, fewer patients experienced secondary negative symptoms (30%) at the end of the observational period.

**Table 3.** Primary and secondary negative symptoms

	Baseline (*n* = 188)	Finale visit^a^ (*n* = 148)
Primary negative symptoms, *n* (%)		
Total	188 (100.0)	148 (100.0)
Affective blunting	175 (93.1)	137 (92.6)
Alogia	100 (53.2)	56 (37.8)
Avolition, apathy	163 (86.7)	91 (61.5)
Anhedonia	154 (81.9)	83 (56.1)
Asociality	143 (76.1)	74 (50.0)
Secondary negative symptoms, *n* (%)		
Total	105 (55.9)	44 (29.7)
Due to positive symptoms	48 (25.5)	21 (14.2)
Due to affective symptoms	69 (36.7)	33 (22.3)
Due to adverse drug reactions	38 (21.2)	9 (6.1)

a 12 months from baseline.

#### Treatment patterns

The treatment approaches of PNS changed slightly throughout the 1-year observational period. At baseline, all patients received pharmacotherapy, 18% antipsychotic monotherapy (M), and 82% polytherapy (P) (Table 4). In addition, 86% of patients received non-pharmacological therapy in the form of supportive psychotherapy (47%), social skills training (13%), and occupational therapy (11%). After 12 months, there was a slight decrease in the number of patients receiving polytherapy (78%) and an increase in non-pharmacological therapies (93%).

**Table 4.** Treatment approaches

	Baseline (*n* = 188)		Final visit^a^ (*n* = 148)	
Type of therapy, *n* (%)				
Pharmacotherapy	188 (100.0)		148 (100.0)	
Antipsychotic monotherapy	34 (18.1)		33 (22.3)	
Antipsychotic polytherapy	154 (81.)		115 (77.7)	
Non–pharmacological therapy	162 (86.2)		138 (93.2)	
Supportive psychotherapy	89 (47.3)		73 (49.3)	
Other types of psychotherapy	10 (5.3)		14 (9.5)	
Social skills training	24 (12.8)		20 (13.5)	
Occupational therapy	21 (11.2)		17 (11.5)	
Electroconvulsive therapy	2 (1.1)		1 (0.7)	
Other	16 (8.5)		13 (8.8)	
Type of antipsychotic, *n* (%)^b^	Mono	Poly	Mono	Poly
Cariprazine	10 (5.3)	135 (71.8)	29 (19.6)	113 (76.4)
Olanzapine	12 (6.4)	60 (31.9)	–	44 (23.4)
Clozapine	5 (2.7)	34 (18.1)	2 (1.4)	31 (20.9)
Quetiapine	3 (1.6)	27 (14.4)	1 (0.7)	17 (11.5)
Aripiprazole	1 (0.5)	22 (11.7)	–	12 (8.1)
Haloperidol	–	23 (12.2)	–	11 (7.4)
Flupentixol	1 (0.5)	17 (9.0)	–	8 (5.4)
Risperidone	1 (0.5)	13 (6.9)	–	7 (4.7)
Paliperidone	1 (0.5)	10 (5.3)	1 (0.7)	7 (4.7)
Reason for stopping antipsychotic treatment, *n* (%)				
Attenuation	1 (0.5)			
Anxiety	2 (1.1)			
Akathisia	3 (1.6)			
Extra–pyramidal symptoms	3 (1.6)			
Insomnia	3 (1.6)			
Other	5 (2.7)			

a 12 months from baseline.

b Taken by more than 5% of patients.

**Figure 1.**
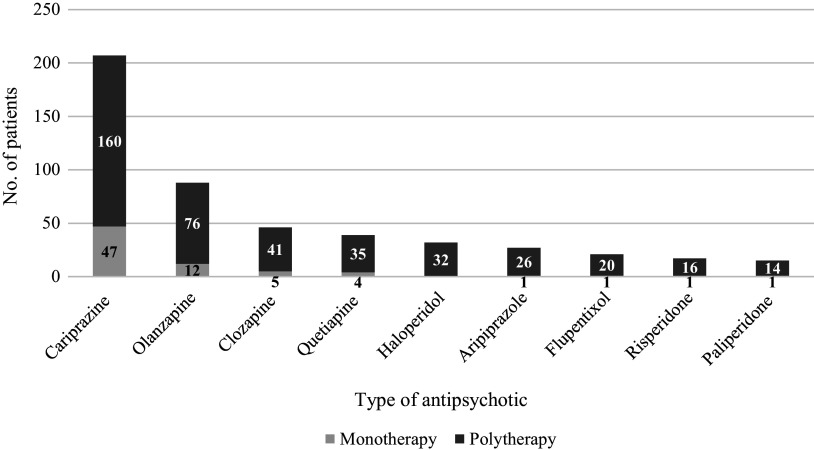
Number of patients taking different types of antipsychotics throughout the observational period.* *Patients taking multiple medications are counted at each drug; drugs with multiple occurrences within a patient are counted only once.

Regarding the specific type of antipsychotics, cariprazine (M: 5%, P: 72%), olanzapine (M: 6%, P: 32%), clozapine (M: 3%, P: 18%), and quetiapine (M: 2%, P: 14%) were prescribed most at baseline. At the final visit, there was an increase in the proportion of patients receiving cariprazine monotherapy (20%) and polytherapy (76%), as well as clozapine polytherapy (21%), while those who received olanzapine (M: 0%, P: 23%) and quetiapine (M: 1%, P: 12%) decreased. All in all, throughout the 1-year period, over 200 patients received cariprazine as either monotherapy or polytherapy, 88 received olanzapine, 46 received clozapine, 39 received quetiapine, 32 received haloperidol, 26 received aripiprazole, 20 received flupentixol, 16 received risperidone, and 14 received paliperidone (Figure 1). The most common reason for stopping any antipsychotic treatment was akathisia, extra-pyramidal symptoms, and insomnia (1.6%) (Table 4).

### Effectiveness of treatment

**Figure 2.**
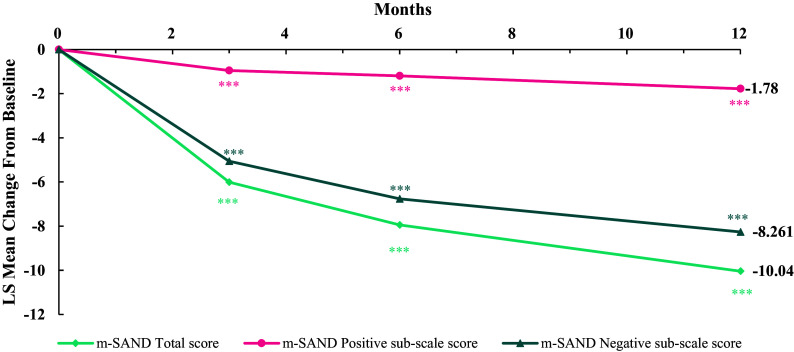
Mean change from baseline in m-SAND total, positive subscale, and negative subscale scores by months. ***p-value <0.0001.

**Figure 3.**
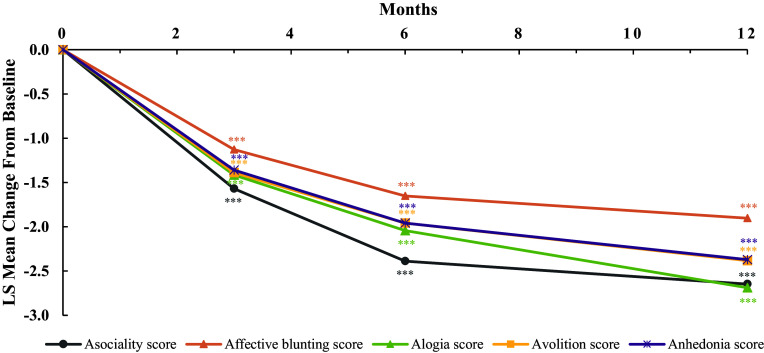
Mean change from baseline in SNS subscores by months. ***p-value <0.0001.

**Figure 4.**
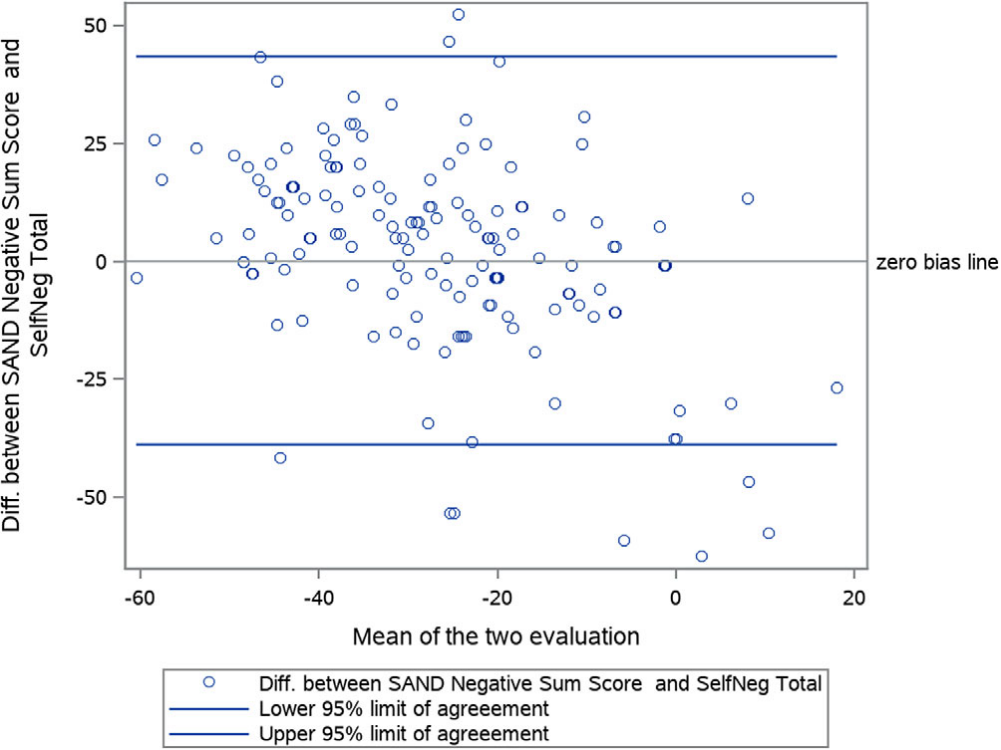
Bland–Altman agreement plot: difference between SAND negative subscore and SNS total score (or change) versus their average scores are expressed as percentage of the corresponding max value.

The mean m-SAND score at baseline was 23.6 with an average 4.6 score on the m-SAND-P subscale and 19.1 on the m-SAND-N subscale (Table 5). A statistically significant 10-point LS mean change from baseline was observed at the end of the observational period on the m-SAND total score with an effect size (ES) of -2.5. The change from baseline was statistically significant from the first visit onwards (Figure 2). In terms of the two subscales, both m-SAND-P (LS mean change: -1.8, p-value <0.0001, ES: -1.6) and m-SAND-N (LS mean change: -8.3, p-value <0.0001, ES: -2.4) changed significantly over the 12 months. Importantly, patients also reported their negative symptoms to have improved as measured by the SNS (LS mean change -12-point in the SNS total score, p-value <0.0001, ES: -1.7) with significant improvement in all five subdomains from the first visit onwards (Figure 3). When comparing the views of patients versus doctors at baseline, patients rated alogia and avolition to be the most severe (based on the SNS), while doctors found affective blunting and then avolition to be the most problematic (based on the m-SAND-N). By the end of the observational period, patients had the highest self-reported scores in avolition and affective blunting. Similarly, physicians rated blunted affect, avolition, and anhedonia to be the most severe. These similarities between the ratings by the patients and doctors are confirmed by a Bland–Altman agreement plot as well, which shows that the difference between the mean changes from baseline to final visit in the SNS and SAND-N lies within the 95% confidence interval around the zero-bias line with doctors reporting a slightly greater improvement compared to patients in negative symptoms (Figure 4). Furthermore, according to the CGI-S scale, the participants were moderately ill at baseline (mean score: 4.3) and mildly ill at the end of the observational period (mean score: 3.0). This detected change was also significant (LS mean change: -1.3, p-value <0.0001, ES: -1.5). Indeed, the mean CGI-I score was 2.2 at the end of study, meaning much improvement. Finally, 54.3% of patients manifested disabilities according to the total PSP scores (scores between 31 and 70) and 45.7% poor functioning (scores under 30) at baseline (Table 5). By the end of the observational period, this changed to 92.6% “manifest disabilities” and only 7.4% “functioning is poor.” This was reflected on the subscales as well where statistically significant change was detected in all categories: socially useful activities (LS mean change: -1.4, p-value <0.0001, ES: -1.5), personal and social relationships (LS mean change: -1.7, p-value <0.0001, ES: -2.0), self-care (LS mean change: -1.5, p-value <0.0001, ES: -1.6), and disturbing and aggressive behaviour (LS mean change: -0.9, p-value <0.0001, ES: -1.9).

**Table 5.** Effectiveness of treatment

	Baseline mean (SD)	Final visit mean (SD)	LS mean change (SE)	ES
m–SAND total	23.6 (5.0)	13.8 (4.4)	−10.0 (0.33)^***^	−2.5
m–SAND–P	4.6 (2.2)	2.9 (1.3)	−1.8 (0.09)^***^	−1.6
Hallucinations	2.1 (1.2)	1.4 (0.7)	−0.7 (0.05)^***^	−1.2
Delusions	2.5 (1.3)	1.5 (0.8)	−1.1 (0.06)^***^	−1.5
m–SAND–N	19.1 (3.8)	11.0 (3.7)	−8.3 (0.28)^***^	−2.4
Anhedonia	4.0 (1.0)	2.2 (1.0)	−1.8 (0.08)^***^	−1.9
Affective blunting	4.3 (0.9)	2.7 (0.9)	−1.6 (0.07)^***^	−1.9
Avolition, apathy	4.2 (1.0)	2.3 (1.0)	−1.9 (0.07)^***^	−2.1
Alogia	2.9 (1.4)	1.8 (0.9)	−1.2 (0.06)^***^	−1.6
Asociality	3.6 (1.3)	1.9 (1.0)	−1.7 (0.07)^***^	−1.9
SNS total	27.4 (7.3)	15.4 (7.1)	−12.0 (0.56)^***^	−1.7
Asociality/items 1–4	5.5 (2.1)	2.9 (1.7)	−2.7 (0.13)^***^	−1.6
Affective blunting/items 5–8	5.2 (1.7)	3.2 (1.5)	−1.9 (0.11)^***^	−1.3
Alogia/items 9–12	5.7 (1.9)	3.1 (1.7)	−2.7 (0.13)^***^	−1.6
Avolition/items 13–16	5.7 (2.0)	3.3 (1.8)	−2.4 (0.14)^***^	−1.4
Anhedonia/items 17–20	5.3 (1.9)	2.9 (1.6)	−2.4 (0.12)^***^	−1.6
CGI–I	–	2.2 (0.8)	–	–
CGI–S	4.3 (1.1)	3.0 (1.0)	−1.31 (0.07)^***^	−1.5
PSP				
Socially useful activities	3.9 (1.1)	2.6 (1.0)	−1.35 (0.07)^***^	−1.5
Personal and social relationships	4.2 (1.0)	2.5 (0.9)	−1.70 (0.07)^***^	−2.0
Self–care	3.3 (1.0)	1.9 (1.0)	−1.52 (0.07)^***^	−1.6
Disturbing and aggressive behaviour	2.0 (1.3)	1.2 (0.5)	−0.90 (0.4)^***^	−1.9

PSP total	Baseline *n* (%)	Final visit *n* (%)		
Only mild difficulties (100–70)	0 (0.0)	0 (0.0)		
Manifest disabilities (70–31)	102 (54.3)	137 (92.6)		
Functioning is poor (30–0)	86 (45.7)	11 (7.4)		

Abbreviations: CGI-I, Clinical Global Impressions – Improvement; CGI-S, Clinical Global Impressions – Severity; ES, effect size; LS, least squares; PSP, the Personal and Social Performance; m-SAND, modified Short Assessment of Negative Domains; m-SAND-N, modified Short Assessment of Negative Domains – negative symptom subscale; m-SAND-P, modified Short Assessment of Negative Domains – positive symptom subscale; SNS, Self-evaluation of Negative Symptoms; SD, standard deviation; SE, standard error.

* *p*-value <0.05.

** *p*-value <0.001.

*** *p*-value <0.0001.

## Discussion

This was the first outpatient, longitudinal, prospective, multicentric cohort study in Slovakia that focused specifically on patients with schizophrenia and predominant negative symptoms. The aim was to do an epidemiologic assessment of the characteristics of negative symptoms, functionality status, disorder insight, and treatment patterns in this patient population throughout a 1-year observational period, along with evaluating the effectiveness of treatment approaches.

According to the results, throughout the 1-year observational period, there has been a significant improvement in all negative symptom domains. Importantly, this positive change was observed by both physicians and patients. As articulated in the most recent guidance by the EPA, including self-report measures is encouraged in negative symptom studies as they can further complement the observer-rated scales when assessing negative symptoms of schizophrenia [17]. In the present case, results based on the SNS and the m-SAND-N scales indicated an agreement between patients and doctors regarding the changes in negative symptoms and highlighted some slight differences in terms of what subdomains of the negative construct are most affected. This comparison was only possible since the SNS and m-SAND-N scales measure the same negative symptom subdomains, the 5As (anhedonia, affective blunting, avolition, alogia, and asociality). It is important to note however that one negative symptom, blunted affect (the decreased expression of emotion), seemed to be the most difficult to treat since, both at baseline and at final visit, 93% of patients were described to exhibit it. Indeed, blunted affect is often unresponsive to treatment and is difficult to measure via rating scales as they are relatively insensitive to change [24]. Nonetheless, according to both the SNS and m-SAND-N scales, the severity of blunted affect decreased significantly, suggesting that some improvement is still possible.

By the end of the study, patients also improved in their functioning, with fewer patients being unemployed and more working occasionally and significant changes in the PSP scores. This is not surprising given the fact that negative symptoms are known to impact everyday functioning [7], and numerous studies reported a link between greater negative symptoms and reduced work functioning [25, 26]. It is important to note however that even though there had been a reduction in the unemployment status, the proportion of patients being unemployed was still higher than what was reported in a study by Szkultecka-Dębek et al. in 2016 (53% vs. 14%) [5]. Additionally, while Szkultecka-Dębek et al. reported 63% of Slovakian patients with schizophrenia to live on disability pension or retirement or employed on sick leave, in the current study, 84% had a disability due to psychiatric illness. Both aspects might be explained by the fact that the participants in the former study were not patients with PNS specifically. In terms of disability status, no improvement was found as the frequency of patients being disabled due to psychiatric illness increased. It is important to note however that in most social care systems [27, 28], schizophrenia is recognized as a qualified condition for disability benefits. Therefore, improvements in overall functioning due to successful treatment does not necessarily translate into a decline of financial support needed that is associated with disability status.

Besides employment and disability status, there was a change in the patients’ insight as well. Insight is defined as “the patient’s capacity to acknowledge some awareness of having an illness” [29] and has also been repeatedly reported to be associated with negative symptoms [30]. For instance, Kemp and Lambert found a correlation between negative symptoms and insight in subjects who improved with treatment [31]. This also seemed to be the case in the present study where alongside the improvement in negative symptoms, the proportion of patients with full insight doubled (from 20% to 45%) and the number of participants with no insight declined.

In terms of typical treatment approaches in Slovakia, the present study showed that most patients received combination therapy (78% at final visit). Although it is not recommended by guidelines, polytherapy is quite common in everyday clinical practice [32]. Indeed, in a survey conducted in five European countries, polypharmacy rates were reported to increase from 19% to 27% between 2000 and 2015 [33]. Interestingly, various studies underline the superiority of polypharmacy compared to monotherapy, especially on parameters such as rehospitalization rates [34] or total symptom reduction [35]. In fact, clozapine combined with a D_2_ partial agonist antipsychotic medication was associated with the lowest risk of rehospitalization even compared to clozapine monotherapy [34], the gold standard in treatment-resistant schizophrenia. Similarly, the most often used augmentation strategy in the present study was an atypical antipsychotic and cariprazine. This might be related to the unique mechanism of action of cariprazine and its efficacy on negative symptoms [15, 22, 36]. Additionally, recent evidence also endorsed the augmentation strategy of clozapine with cariprazine [37–40] by reporting good tolerability and safety, as well as further reduction in negative symptoms. [14, 41]

Cariprazine was the most popular medication as monotherapy too with 20% of participants being on cariprazine treatment alone at final visit. This is in line with the treatment algorithm by Cerveri et al [42]. The results also provide confirmation to the claim that this algorithm has been adapted in Slovakia [16]. Rancans et al. conducted a 16-week observational study on the effectiveness of cariprazine with PNS patients as well [22]. The results of the observational study are comparable to this cohort study; participants in both studies were patients with PNS with a baseline CGI of moderate severity (present study: 4.3, Rancans et al.: 4.4), and the primary outcome measure was the SAND [22] and the m-SAND [22]. In addition, it also shows that improvement in this symptom domain is slower and continuous with no plateauing of improvement at any point of the 12 months.

The present study has multiple limitations. First, due to the nature of the study design, results have limited internal validity due to probable selection and different biases such as observer bias, inter-rater bias, information bias, and measurement bias [43, 44]. Internal validity plays a crucial role in establishing the effectiveness of a treatment; it ensures that the observed effects are directly attributable to the treatment itself, rather than being influenced by other external factors [44]. However, the primary objective of this study was not to establish efficacy, but to understand the typical treatment and symptom patterns of patients with schizophrenia and PNS in Slovakia. The second limitation is that the primary outcome measure of the study was a non-validated scale. Nonetheless, using standardized scales in real-life settings is often not feasible, and thus, to better mimic real-life settings, the m-SAND was utilized [22]. Although the m-SAND scale is not validated, it is based on the CGI-S scale, which is known to have good inter-rater reliability among clinicians [21, 22]. Future research should aim to further investigate what combinations are the most effective in improving PNS as we have seen that besides cariprazine, most patients took an additional antipsychotic medication as well.

## Conclusion

In conclusion, with the right treatment strategy, it is possible to improve PNS as well as everyday functioning in outpatients with schizophrenia. One of the most used antipsychotic medications in this patient population was cariprazine, which had been utilized both alone and in combination with other antipsychotics. This strategy is in line with the treatment algorithm for negative symptoms in schizophrenia suggested by Cerveri et al., which recommends cariprazine as a first-line medication for the treatment of negative symptoms [42]. It is also important to note that the improvement in negative symptoms was continuous throughout the one-year observation with no plateauing at any point, suggesting that patience is key in negative symptom treatment.

## Supporting information

Dragasek et al. supplementary material Dragasek et al. supplementary material

## Data Availability

The data that support the findings of this study are available from the corresponding author, Z. B. Dombi, upon reasonable request.

## Supplementary material

The supplementary material for this article can be found at http://doi.org/10.1192/j.eurpsy.2024.1757.
