# Supplementary material for: The management of patients with predominant negative symptoms in Slovakia: A 1-year longitudinal, prospective, multicentric cohort study
Source: Eur Psychiatry. 2024 May 23;67(1):e44. doi: 10.1192/j.eurpsy.2024.1757 (PMC11441340; doi:10.1192/j.eurpsy.2024.1757)
Supplement: Dragasek et al. supplementary material [file S0924933824017577sup001.docx]

Supplement

The modified SAND scale.

| **Hallucinations**  Perceptions in any sensory modality in the absence of external stimulus that has qualities of real perception. | | | | | |
| --- | --- | --- | --- | --- | --- |
| Not observed | Mild | Moderate | Moderately severe | Severe | Extreme |
| **Delusions**  A delusion is a fixed belief that is not amenable to change in light of conflicting evidence. | | | | | |
| Not observed | Mild | Moderate | Moderately severe | Severe | Extreme |
| **Anhedonia**  The diminished capacity to experience pleasant emotions. | | | | | |
| Not observed | Mild | Moderate | Moderately severe | Severe | Extreme |
| **Blunted affect**  A decrease in the observed expression of emotion, i.e. facial and vocal expression, and expressive gestures. | | | | | |
| Not observed | Mild | Moderate | Moderately severe | Severe | Extreme |
| **Avolition / Apathy**  A reduced initiation and persistence of goal-directed activity, refers to the reduction of engagement by the person in activities relevant to work, school, everyday life and hobbies. | | | | | |
| Not observed | Mild | Moderate | Moderately severe | Severe | Extreme |
| **Alogia**  A reduction in the quantity of speech and in its spontaneous elaboration. | | | | | |
| Not observed | Mild | Moderate | Moderately severe | Severe | Extreme |
| **Asociality**  A reduction in social initiative due to decreased interest in forming close relationships with others. | | | | | |
| Not observed | Mild | Moderate | Moderately severe | Severe | Extreme |
| *Definitions based on concepts presented in Marder, S. R., and S. Galderisi. 2017. 'The current conceptualization of negative symptoms in schizophrenia', World Psychiatry, 16: 14-24.* | | | | | |
